# Supplementary material for: Tyrosinase Inhibition and Kinetic Details of Puerol A Having But-2-Enolide Structure from Amorpha fruticosa
Source: Molecules. 2020 May 18;25(10):2344. doi: 10.3390/molecules25102344 (PMC7287670; doi:10.3390/molecules25102344)
Supplement: Supplementary file 1 [file molecules-25-02344-s001.pdf]

Supplementary material

Tyrosinase inhibition and kinetic details of puerol A having but-2-enolide structure from *Amorpha fruticosa*

▶Characterization Data

- Figure S1−S7: NMR and HREIMS data of compound **1**
- Figure S8−14: NMR and HREIMS data of compound **2**
- Figure S15: The HPLC peak of compound **1** and **2**
- Figure S16: The fluorescence quenching spectra of compound **2**
- Figure S17: The B16F10 cell experiment data of compound **1**
- Table 1: ^{1}H and ^{13}C NMR data of compound **1** in MeOH-d_4.
- Table 2: ^{1}H and ^{13}C NMR data of compound **2** in Acetone-d_6

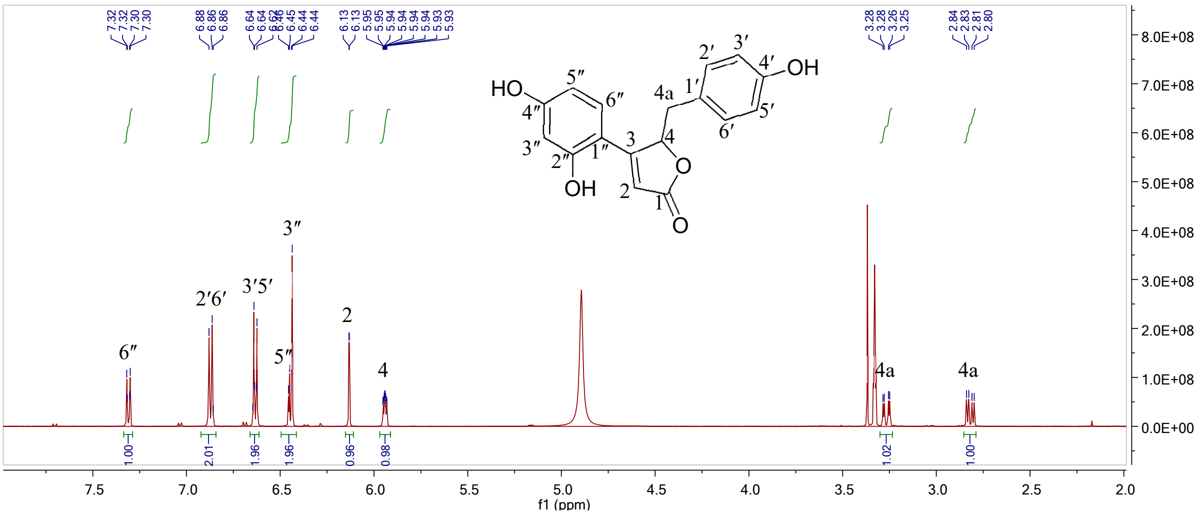

Figure S1. ^{1}H-NMR spectrum of compound **1** (500mHz, MeOH-d_4)

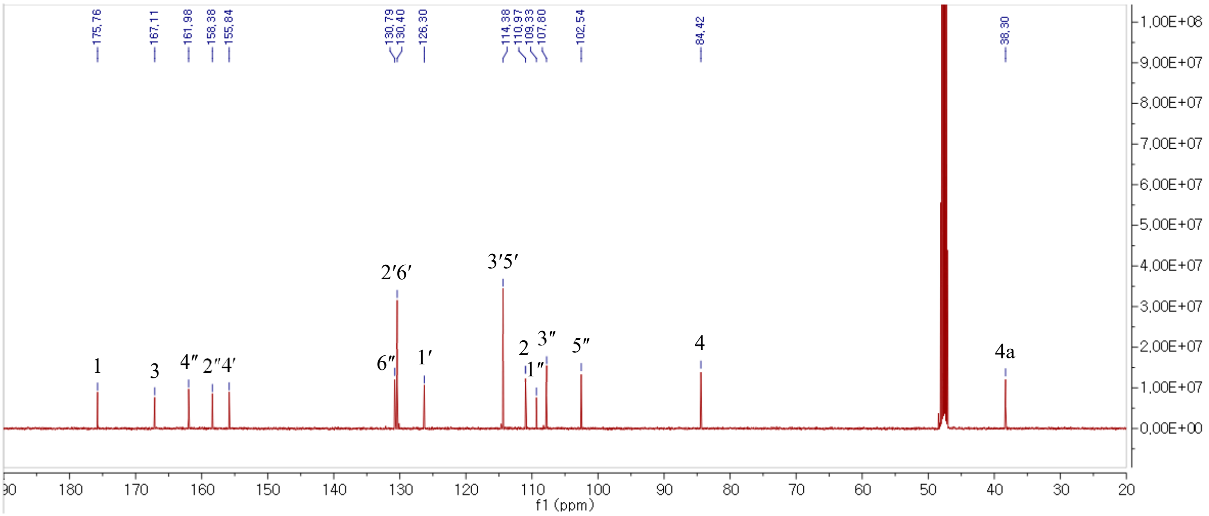

Figure S2. ^{13}C-NMR spectrum of compound **1** (500mHz, MeOH-d_4)

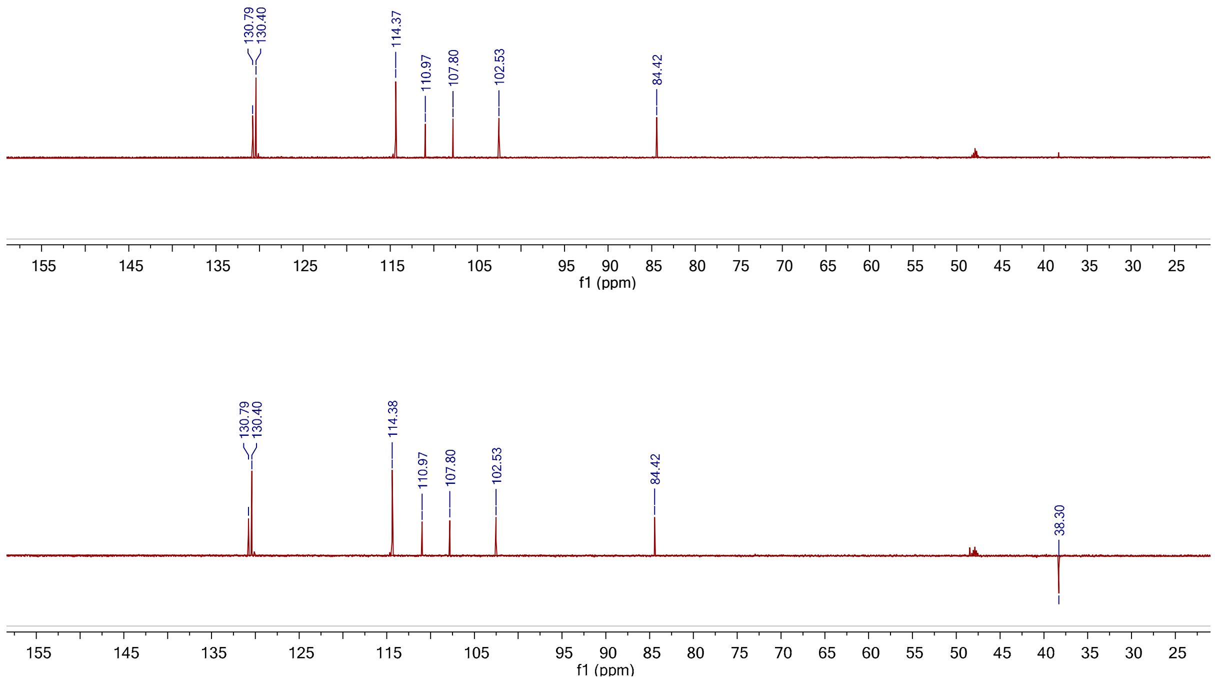

Figure S3. DEPT-90 and -135 spectrum of compound **1.**

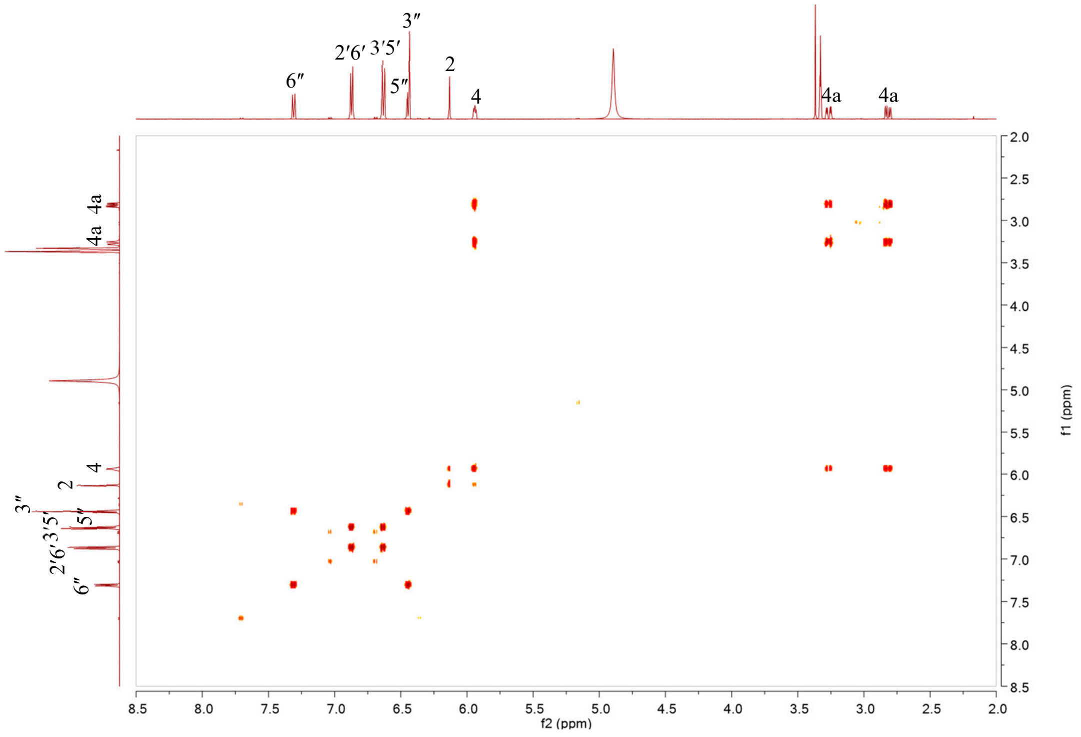

Figure S4. COSY spectrum of compound **1**.

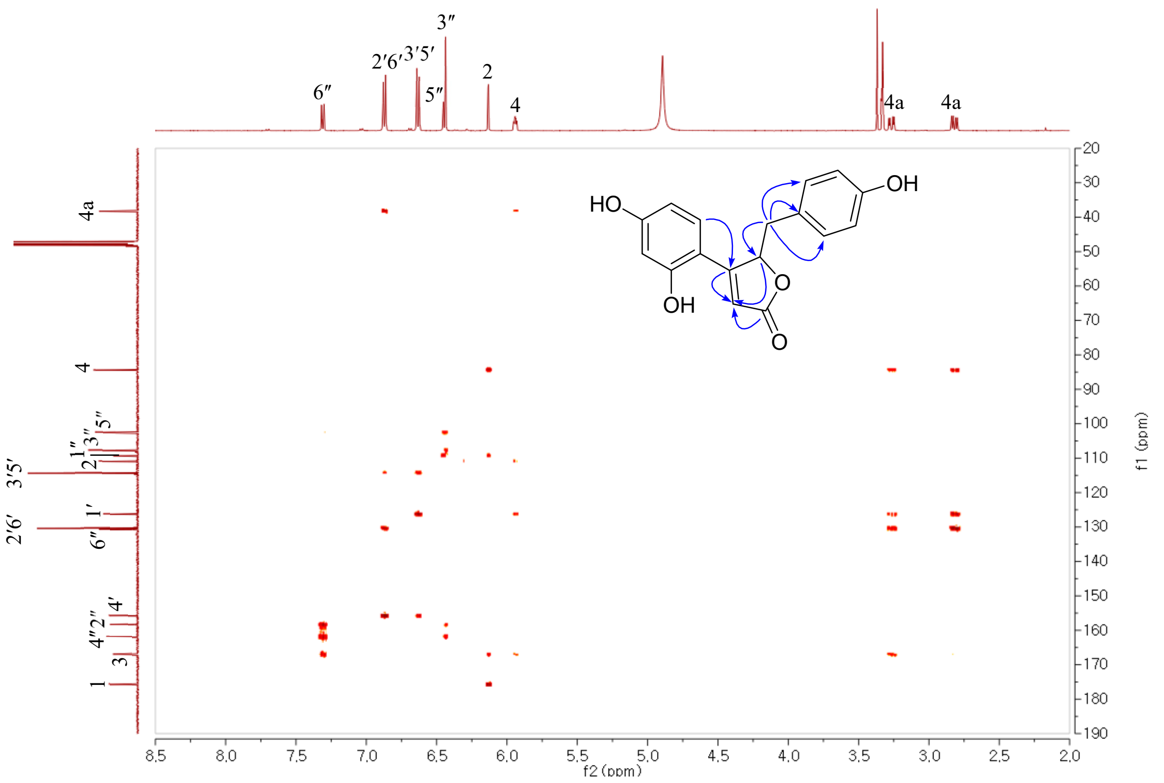

Figure S5. HMBC spectrum of compound **1**.

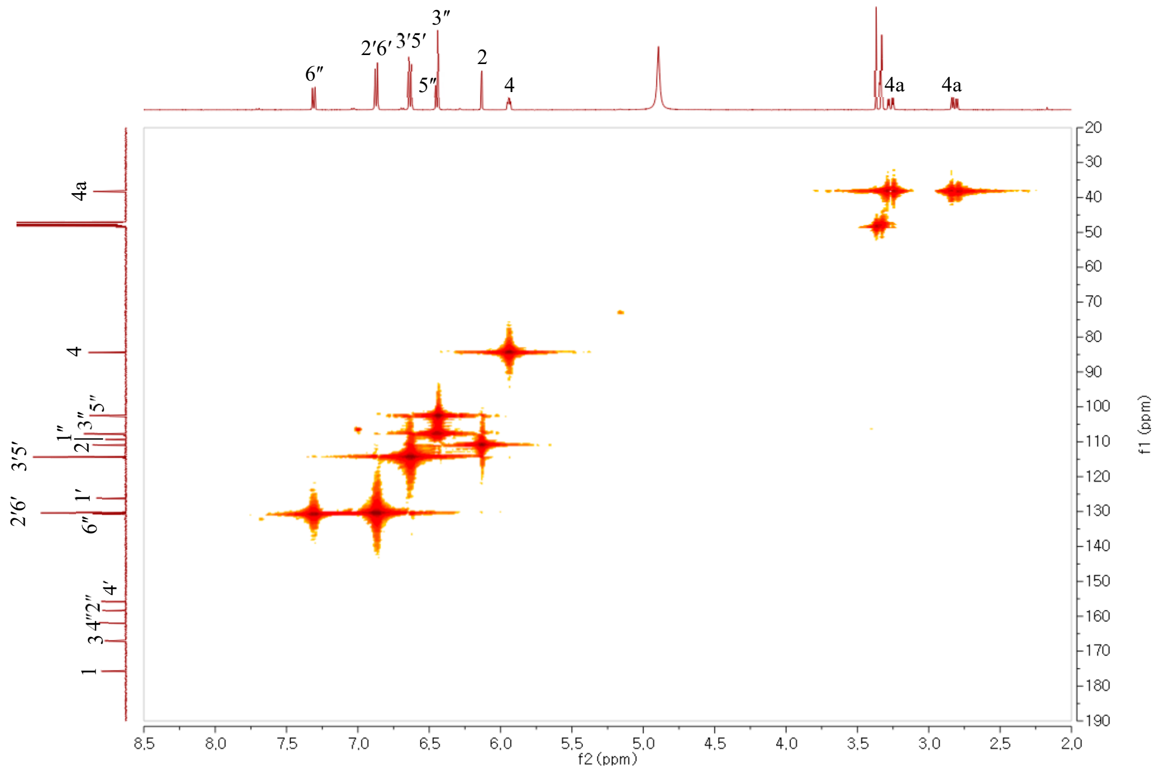

Figure S6. HMQC spectrum of compound **1**.

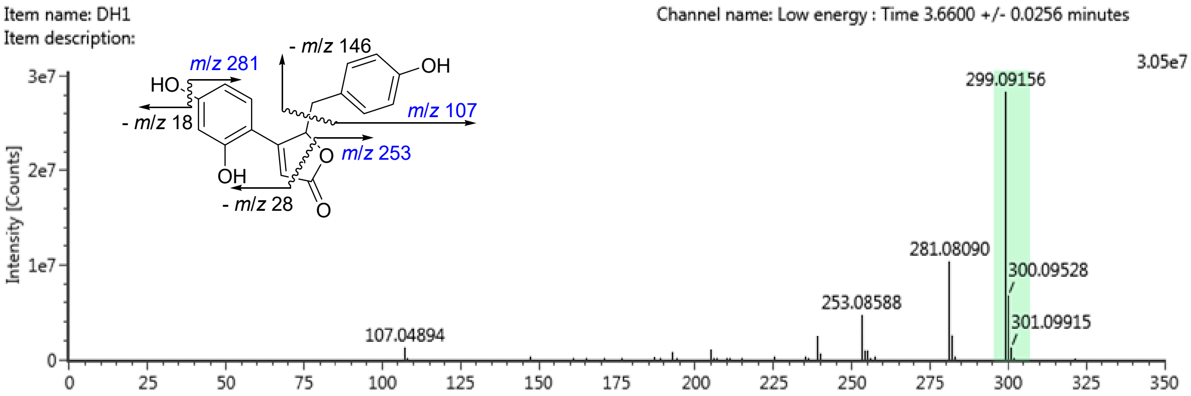

Component name	Identification status	Formula	Neutral mass (Da)	Observed neutral mass (Da)	Observed m/z	Mass error (mDa)	Mass error (ppm)	Observed RT (min)	Detector counts	Adducts
Compound 1	Identified	$C_{17}H_{14}O_5$	298.08412	298.0843	299.0916	0.2	0.5	3.66	4762262	+H

Figure S7. HRESIMS data of compound **1**.

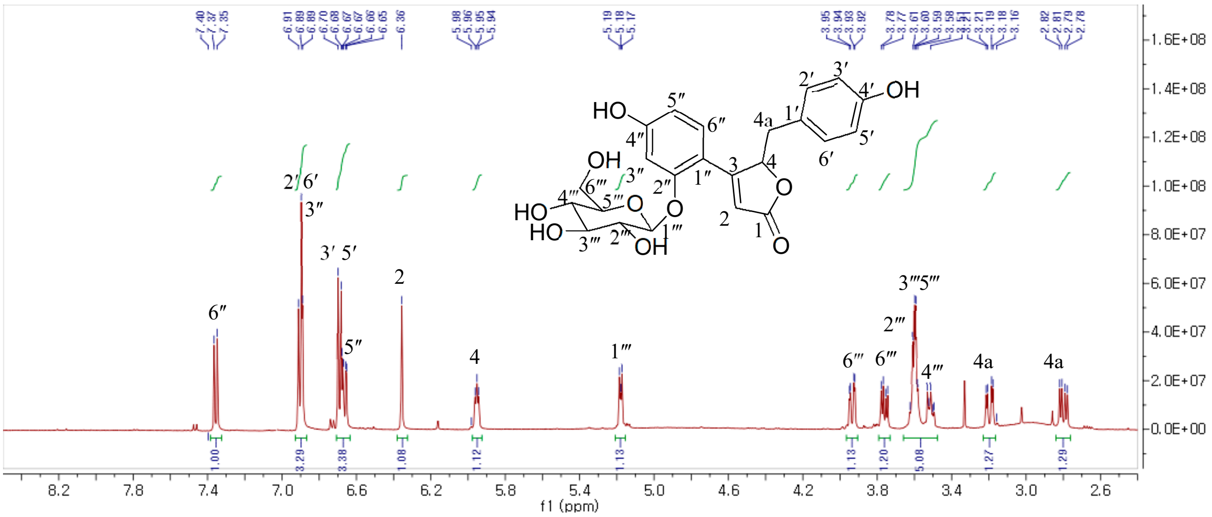

Figure S8. ^{1}H-NMR spectrum of compound **2** (500mHz, Acetone-d_6)

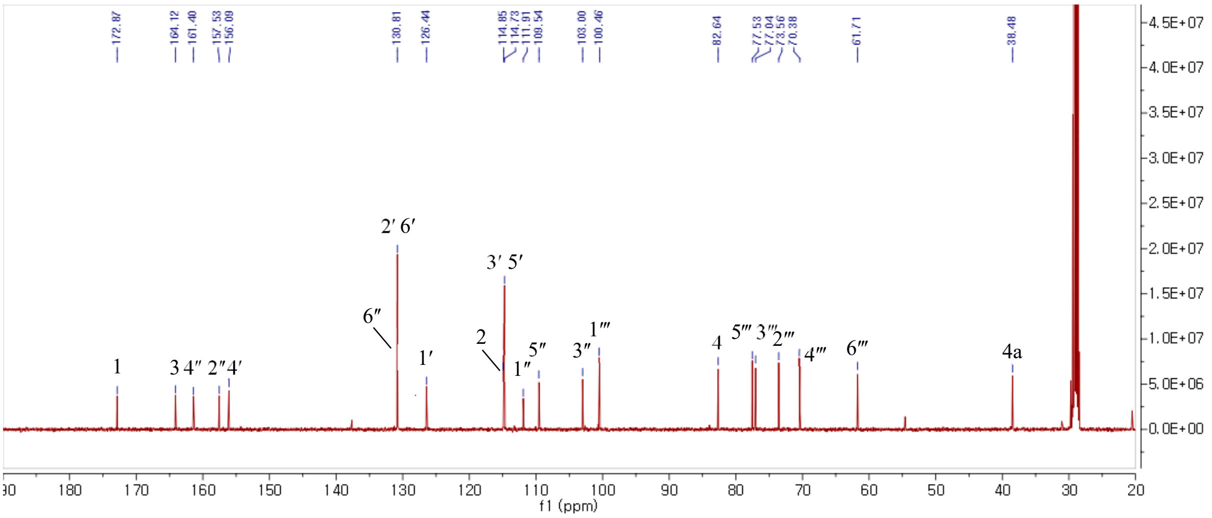

Figure S9. ^{13}C-NMR spectrum of compound **2** (500mHz, Acetone-d_6)

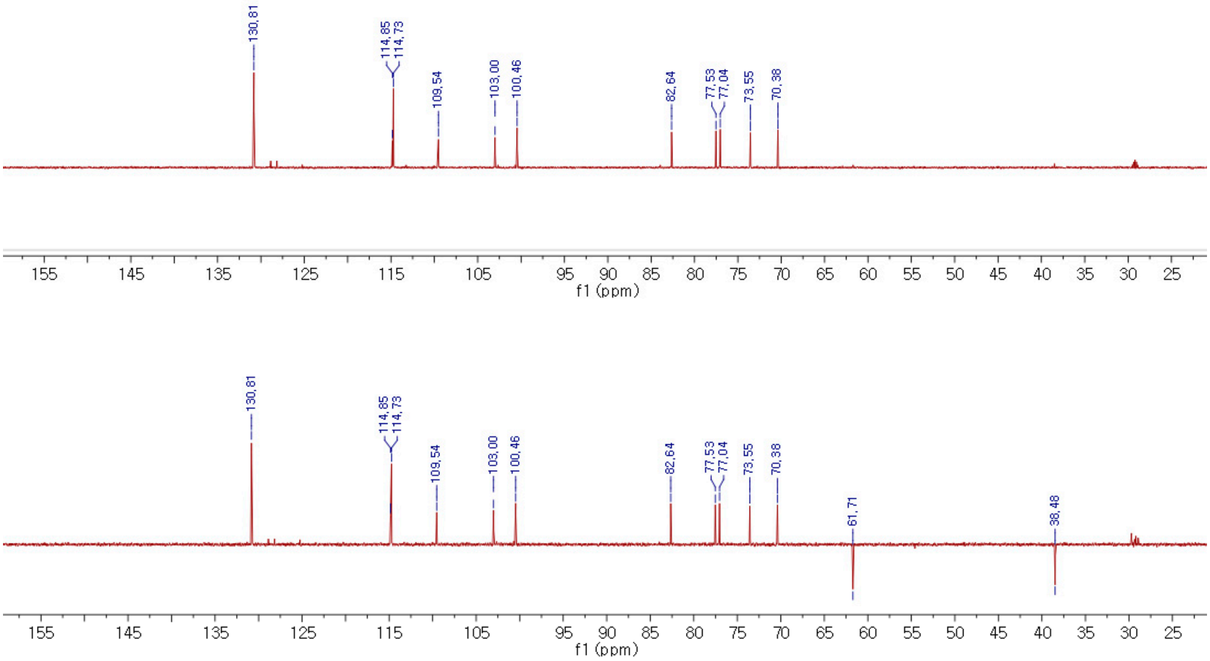

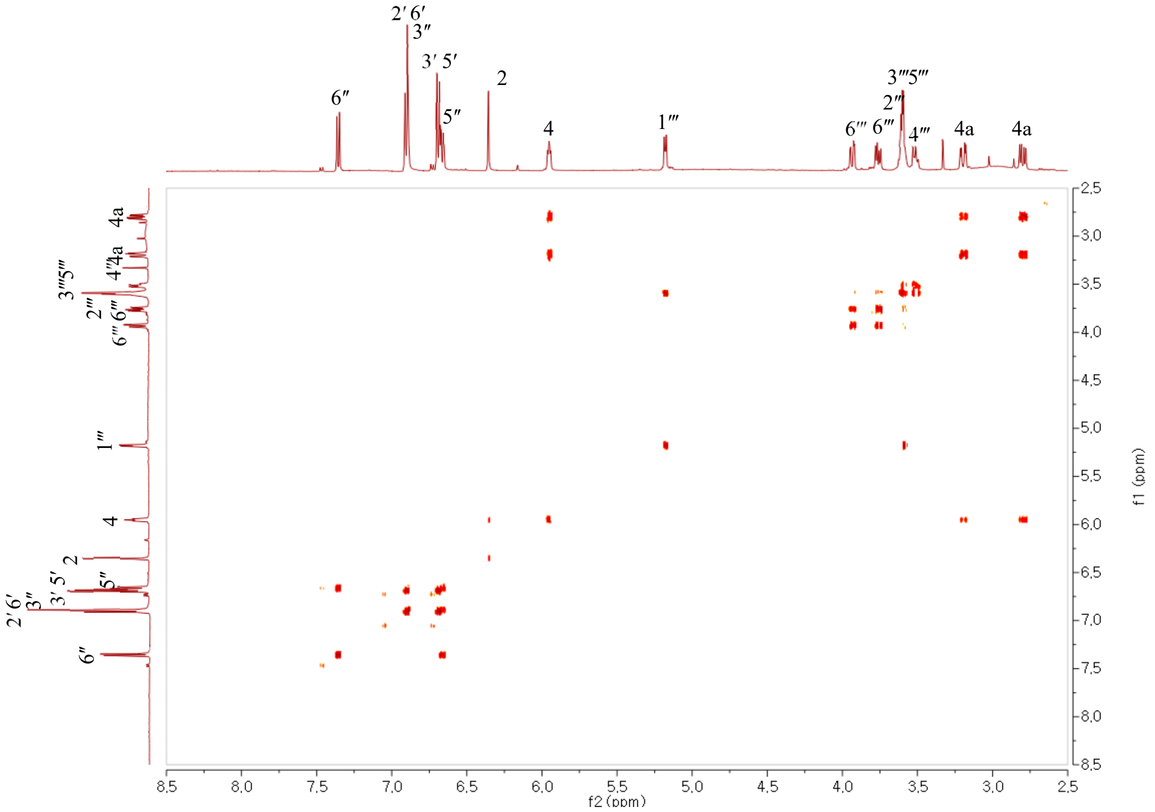

Figure S10. COSY spectrum of compound **2**.

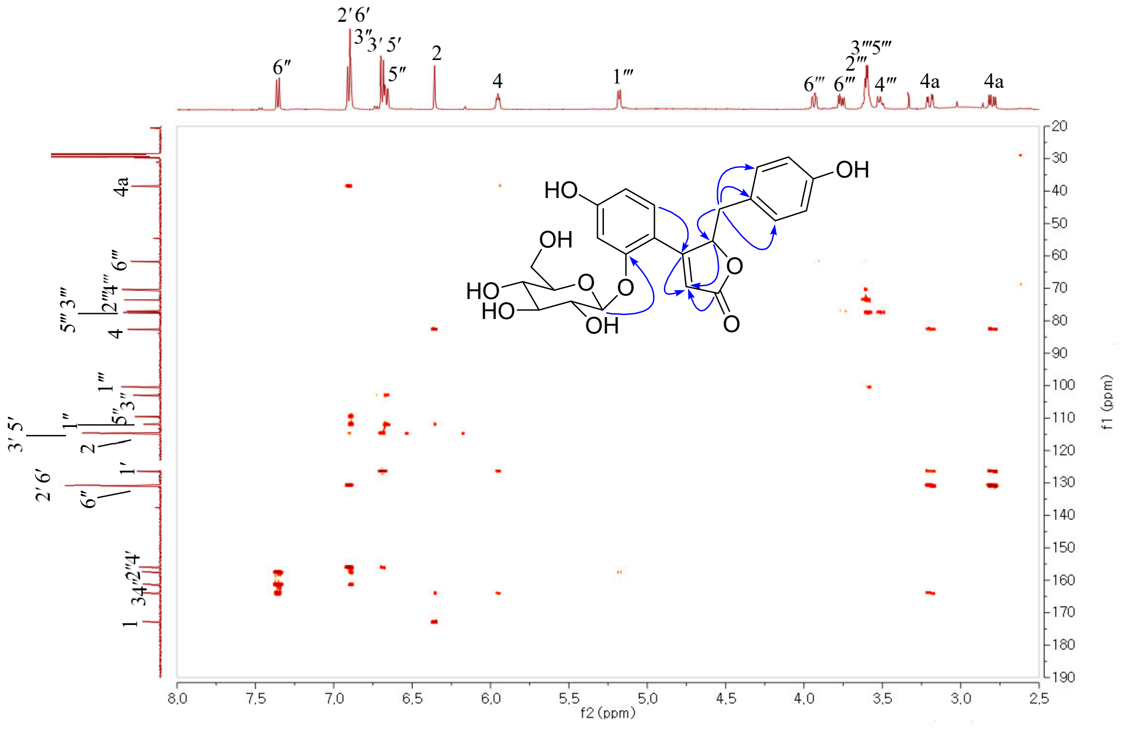

Figure S11. HMBC spectrum of compound **2**

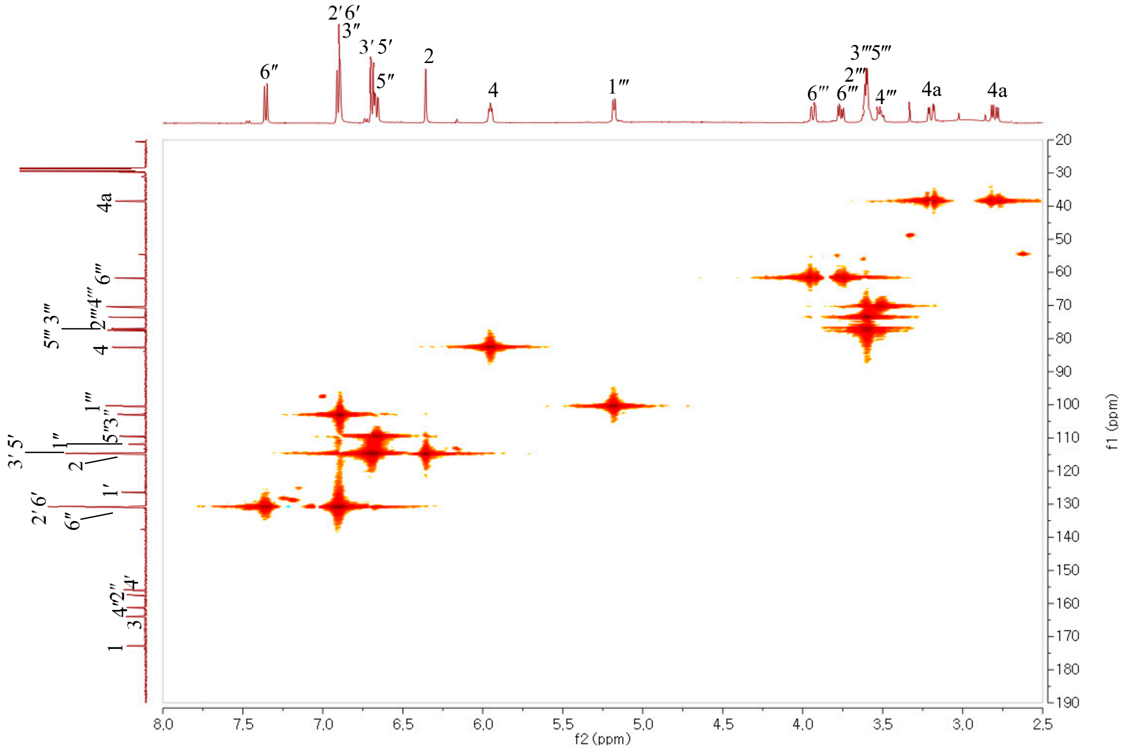

Figure S12. HMQC spectrum of compound **2**.

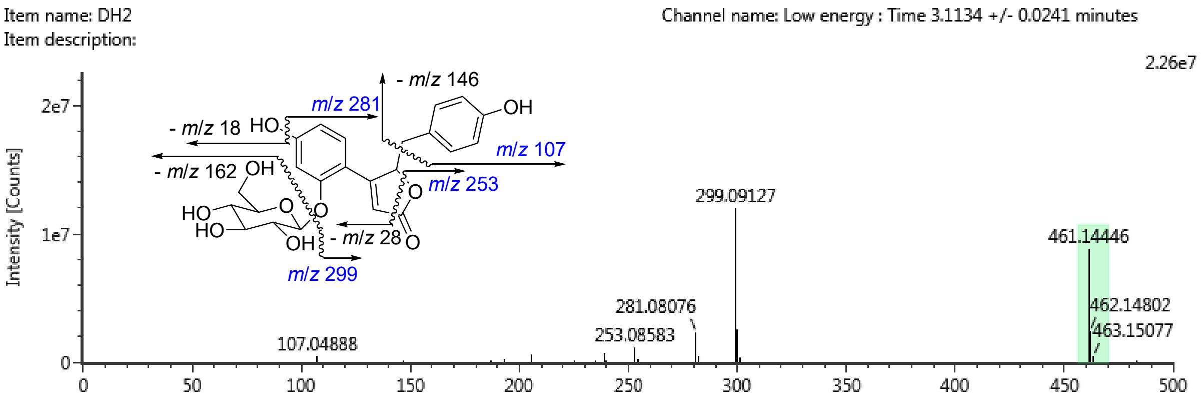

Component name	Identification status	Formula	Neutral mass (Da)	Observed neutral mass (Da)	Observed m/z	Mass error (mDa)	Mass error (ppm)	Observed RT (min)	Detector counts	Adducts
Compound 2	Identified	$C_{23}H_{24}O_{10}$	460.13695	460.1372	461.1445	0.2	0.5	3.11	1940588	+H

Figure S13. HRESIMS data of compound **2**.

(A)

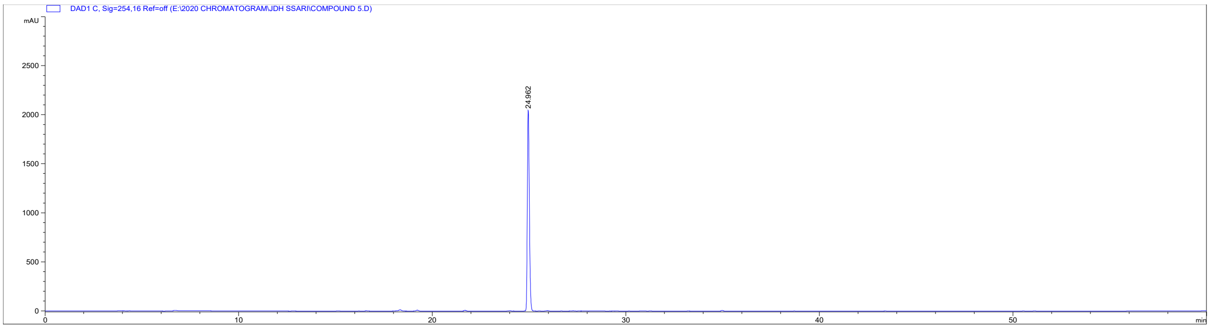

(B)

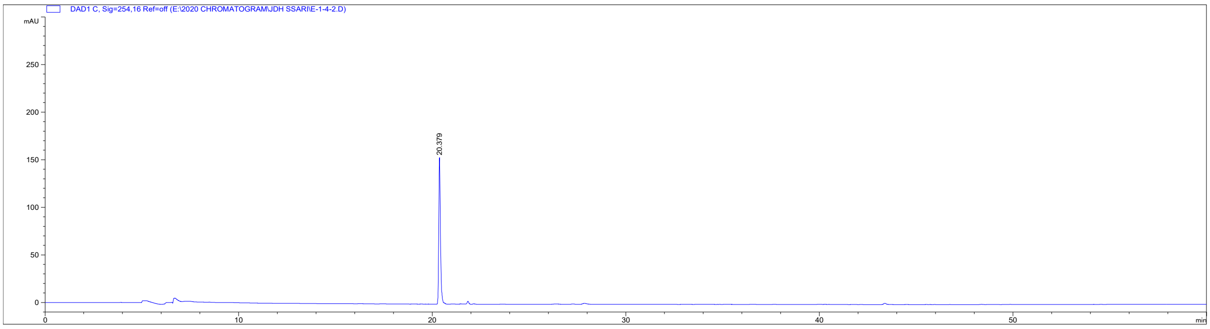

Figure S15. HPLC peak of (A) puerol A and (B) kuzubutenolide A

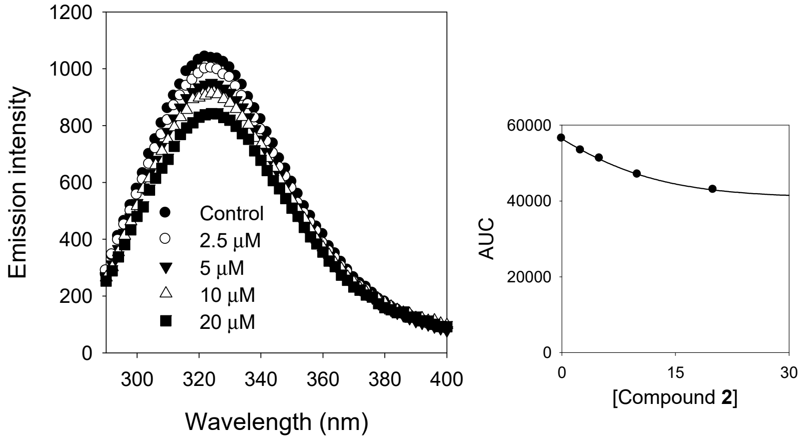

Figure S15. The fluorescence emission spectra of tyrosinase at different concentrations of compound 2 (Inset) Normalized intensities of fluorescence for tyrosinase are shown for compounds **2**.

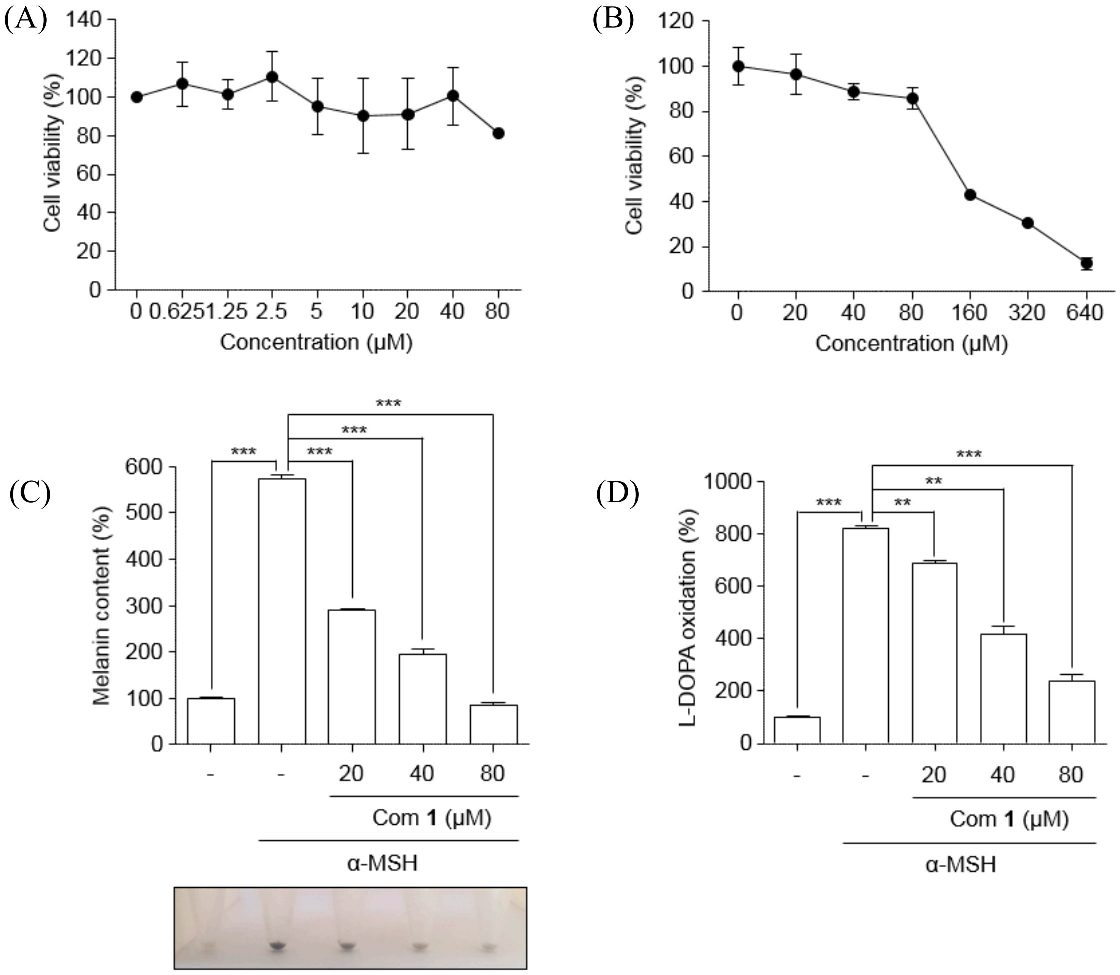

Figure S17. (A and B) Effects of compound **1** on B16F10 melanoma cell viability incubated at each concentration for 48 h. (C) Effect of compound **1** lowering melanin synthesis in B16F10 cells. (D) Effect of compound **1** reducing L-DOPA oxidation in B16F10 cells.

Table S1. ^{1}H NMR and ^{13}C NMR data of compound **1** (500 MHz, MeOD)

Position	δ_H (multi, *J* in Hz)	δ_C (ppm)
1	-	175.7
2	6.13 (1H, d, *J* = 1.1 Hz)	110.9
3	-	167.1
4	5.95 (1H, ddd, *J* = 1.1, 3.5, 5.8 Hz)	84.4
4a	2.80 (1H, dd, *J* = 6.0, 14.5 Hz)<br>3.25 (1H, dd, *J* = 3.5, 14.5 Hz)	38.3
1′	-	126.2
2′	6.86 (2H, d, *J* = 8.5 Hz)	130.4
3′	6.62 (2H, d, *J* = 8.5 Hz)	114.3
4′	-	155.8
5′	6.62 (2H, d, *J* = 8.5 Hz)	114.3
6′	6.86 (2H, d, *J* = 8.5 Hz)	130.4
1″	-	109.3
2″	-	158.3
3″	6.44 (1H, d, *J* = 1.5 Hz)	107.7
4″	-	161.9
5″	6.45 (1H, d, *J* = 2.3 Hz)	102.5
6″	7.30 (1H, dd, *J* = 1.6, 7.4 Hz)	130.7

Table S2. ^{1}H NMR and ^{13}C NMR data of compound **2** (500 MHz, Aceton-d6)

Position	δ_H (multi, *J* in Hz)	δ_C (ppm)
1	-	172.8
2	6.36 (1H, d, *J* = 1.1 Hz)	114.8
3	-	164.1
4	5.95 (1H, ddd, *J* = 1.4, 3.4, 6.4 Hz)	82.6
4a	2.79 (1H, dd, *J* = 6.2, 14.7 Hz)<br>3.19 (1H, dd, *J* = 3.7, 14.6 Hz)	38.4
1′	-	126.4
2′	6.91 (2H, d, *J* = 8.6 Hz)	130.8
3′	6.70 (2H, d, *J* = 8.6 Hz)	114.7
4′	-	156.0
5′	6.70 (2H, d, *J* = 8.6 Hz)	114.7
6′	6.91 (2H, d, *J* = 8.6 Hz)	130.8
1″	-	111.9
2″	-	157.5
3″	6.89 (1H, d, *J* = 1.6 Hz)	103.0
4″	-	161.4
5″	6.67 (1H, dd, *J* = 2.2, 8.6 Hz)	109.5
6″	7.37 (1H, d, *J* = 8.5 Hz)	130.8
1‴	5.18 (1H, t, *J* = 7.1 Hz)	100.4
2‴	3.60 (1H, m)	73.5
3‴	3.58 (1H, m)	77.0
4‴	3.52 (1H, m)	70.3
5‴	3.59 (1H, m)	77.5
6‴	3.93 (1H, m)	61.7
